# Chronic *Toxoplasma gondii* in Nurr1-Null Heterozygous Mice Exacerbates Elevated Open Field Activity

**DOI:** 10.1371/journal.pone.0119280

**Published:** 2015-04-09

**Authors:** Jeffrey B. Eells, Andrea Varela-Stokes, Shirley X. Guo-Ross, Evangel Kummari, Holly M. Smith, Arin D. Cox, David S. Lindsay

**Affiliations:** 1 Department of Basic Sciences, College of Veterinary Medicine, Mississippi State University, Mississippi State, Mississippi, United States of America; 2 Department of Biomedical Sciences & Pathobiology, Virginia–Maryland Regional College of Veterinary Medicine, Virginia Tech, Blacksburg, Virginia, United States of America; University of California, Riverside, UNITED STATES

## Abstract

Latent infection with *Toxoplasma gondii* is common in humans (approximately 30% of the global population) and is a significant risk factor for schizophrenia. Since prevalence of *T*. *gondii* infection is far greater than prevalence of schizophrenia (0.5-1%), genetic risk factors are likely also necessary to contribute to schizophrenia. To test this concept in an animal model, Nurr1-null heterozygous (+/-) mice and wild-type (+/+) mice were evaluate using an emergence test, activity in an open field and with a novel object, response to bobcat urine and prepulse inhibition of the acoustic startle response (PPI) prior to and 6 weeks after infection with *T*. *gondii*. In the emergence test, *T*. *gondii* infection significantly decreased the amount of time spent in the cylinder. *Toxoplasma gondii* infection significantly elevated open field activity in both +/+ and +/- mice but this increase was significantly exacerbated in +/- mice. *T*. *gondii* infection reduced PPI in male +/- mice but this was not statistically significant. Aversion to bobcat urine was abolished by *T*. *gondii* infection in +/+ mice. In female +/- mice, aversion to bobcat urine remained after *T*. *gondii* infection while the male +/- mice showed no aversion to bobcat urine. Antibody titers of infected mice were a critical variable associated with changes in open field activity, such that an inverted U shaped relationship existed between antibody titers and the percent change in open field activity with a significant increase in activity at low and medium antibody titers but no effect at high antibody titers. These data demonstrate that the Nurr1 +/- genotype predisposes mice to *T*. *gondii*-induced alterations in behaviors that involve dopamine neurotransmission and are associated with symptoms of schizophrenia. We propose that these alterations in murine behavior were due to further exacerbation of the altered dopamine neurotransmission in Nurr1 +/- mice.

## Introduction

Schizophrenia is a neuropsychiatric illness that is diagnosed based on positive symptoms such as delusions, paranoia and hallucination and negative symptoms such as blunted emotions. Recent gene wide association studies have identified gene variants that have only a small association with increased risk of schizophrenia [1]. Copy number variations in some genes are associated with a greater risk for schizophrenia; however, these are very rare [2]. A number of environmental and developmental stressors, such as being raised in an urban area, juvenile cannabis use, obstetric complications, maternal infection during pregnancy and infection with *Toxoplasma gondii* have all been shown to increase the risk of developing schizophrenia [3,4]. One prevailing hypothesis is that genetic susceptibility and environmental stressors interact to potentiate the risk of schizophrenia.

*Toxoplasma gondii* is an obligate intracellular protozoan parasite that undergoes sexual reproduction in a cat host, where it is shed as oocysts into the environment through feces. *Toxoplasma gondii* also infects most warm-blooded vertebrates, reproducing asexually during an acute phase that triggers an immune response and the production of antibodies to the parasite [5]. Following the initial immune response, the parasite enters a chronic phase whereby it forms quiescent tissue cysts with bradyzoite stages, primarily in brain and muscle tissues. Globally, the rate of human infection has been estimated at 30%, with rates as high as 80% in some countries [5]. While these infections have been considered benign, evidence is accumulating that suggests that *T*. *gondii* infection alters human behavior and, in some cases, contributes to or exacerbates mental illness [6–8]. Interestingly, slower reaction time, an increase in traffic accidents and associations with specific personality traits were reported in individuals with antibody titers to *T*. *gondii* [8]. High antibody titers to this parasite also increase the incidence of schizophrenia (average odds ratio of ~2.6), increase severity of schizophrenia symptoms [4,9–13] and exacerbate gray matter reductions in schizophrenia patients [14]. Additionally, children born to a mother with high antibody titers have a similar elevated risk for developing schizophrenia [4,15–17] This relatively high risk factor, combined with the high prevalence of this parasite suggests that it could be a substantial contributor to the number of schizophrenia cases [18,19].

One proposed mechanism by which *T*. *gondii* affects behavior is through elevating dopamine neurotransmission. An initial study found elevated tissue dopamine levels (14%) in whole brain of mice after *T*. *gondii* infection [20]. The strongest support for this dopamine hypothesis was provided by the discovery that *T*. *gondii* expresses two tyrosine hydroxylase enzymes that can synthesize DOPA *in vitro* using enzymatic assays and ultimately dopamine *in vivo* based on immunohistochemical labeling of dopamine [21,22]. In addition open field activity, a behavior closely linked with dopamine neurotransmission, is commonly found to be elevated after infection with *T*. *gondii* [23–26]. This hypothesized mechanism is particularly relevant to schizophrenia as elevated subcortical dopamine neurotransmission has been implicated as an important factor in the positive symptoms of schizophrenia and antipsychotics work by blocking the dopamine D2 receptor [27–29].

Although infection with *T*. *gondii* does not cause schizophrenia itself, as evidenced by much greater rates of *T*. *gondii* infection (~30%) compared to schizophrenia (~1%), *T*. *gondii* infection could serve as a perturbation that combines with a genetic predisposition to lead to schizophrenia. Nurr1 (NR4A2) is an orphan nuclear receptor that is essential for the development and continued survival of mesencephalic dopamine neurons [30–33]. This receptor is implicated as a potential contributor to the development of schizophrenia as rare mutations in Nurr1 have been reported in schizophrenia patients [34,35] and were associated with attention deficits in schizophrenia patients [36]. Although the homozygous deletion of Nurr1 is lethal at birth, Nurr1-null heterozygous (+/-) mice survive normally. The heterozygous genotype, however, causes alterations in mesoaccumbens and mesocortical dopamine levels and elevated open field activity [37]. Because of these changes, the Nurr1 +/- mice have been investigated as a model for schizophrenia-related behaviors [37–40]. Furthermore, these mice are sensitive to the developmental stressor of post-weaning isolation, an experimental treatment used as a model for early life stressors that contribute to the risk of schizophrenia. Post-weaning isolation of +/- mice specifically disrupted sensorimotor gating as measured by prepulse inhibition of the acoustic startle response (PPI), a parameter that is also disrupted in patients with schizophrenia and correlates with positive symptoms [37–47]. Additionally, these mice had elevated amphetamine-stimulated dopamine release in the shell of the nucleus accumbens, a parameter that is also similar to what has been found in patients with schizophrenia [48]. Therefore, these mice represent a condition of susceptibility to alterations in a behavior disrupted in schizophrenia that requires an environmental insult.

Based on the observations that the Nurr1 +/- genotype alters dopamine neurotransmission and open field activity and infection with *T*. *gondii* is proposed to alter dopamine neurotransmission, the goals of the current study were to determine how *T*. *gondii* infection interacts with the +/- genotype to affect dopamine related behaviors. Our results demonstrate a particular susceptibility of +/- mice to the behavioral effects of *T*. *gondii* infection, specifically elevated open field activity and a trend toward disrupted sensorimotor gating. Additionally, we demonstrate an inverted U relationship between antibody titers and the effect on open field activity.

## Materials and Methods

### Ethics Statement

All procedures were performed in accordance with the National Institutes of Health Guide for the Care and Use of Laboratory Animals, and study protocols were approved by the Institutional Animal Care and Use Committee at Mississippi State University. All animals used in this project were housed in the AAALAC accredited facilities of the College of Veterinary Medicine, Mississippi State University. The individual room temperatures were maintained between 18–22°C with food and water *ad libitum*. Care of the mice was overseen by a laboratory animal veterinarian.

### Animals

Animals were housed at Mississippi State University in an AAALAC accredited facility on 12 hour light/dark cycle with food and water available *ad libitum*. Nurr1 +/- and wild-type (+/+) mice used were obtained from a colony currently bred at Mississippi State University and originally produced in the laboratory of Dr. Vera Nikodem at the National Institute for Diabetes and Digestive and Kidney Diseases [30]. Mice were genotyped as previously described to distinguish +/- and +/+ mice [30]. The Nurr1 +/- mice were breed into a C57BL6 background. At 19–21 days of age, mice were weaned and housed in groups of 3-5/cage. All mice were housed in cages with steel grid lids and all cages were located in the same room.

### Behavioral tests

The following behavior tests were performed on 120-day old Nurr1 +/- and +/+ mice prior to and six-weeks after infection on separate days with each test period 3–4 days apart: (1) PPI; (2) Emergence, open field and novel object measured sequentially in 20 min intervals; 3) open field and bobcat urine response measured sequentially in 20 min intervals. The PPI, emergence, open field, and novel object and bobcat urine tests were all performed between 0800 and 1200 on different days with each test 3–4 days apart. Body weights were recorded following the PPI test. After initial behavior testing, mice were injected subcutaneously with 200 μl Hank’s Buffered Salt Solution (HBSS) containing 1000 tachyzoites (*T*. *gondii* ME49) or HBSS without tachyzoites. *Toxoplasma gondii* ME49 is a type II strain and was chosen for these studies because type II strains are most commonly reported in human *T*. *gondii* infections in the United States. Six weeks post-injection, behavior testing was repeated. After the completion of testing, the mice were euthanized by CO_2_ asphyxiation. Trunk blood samples were collected from sacrificed mice into Vacutainer tubes with heparin. Blood samples were centrifuged at 2000 x g for 5 min and plasma collected for serological testing. *Toxoplasma gondii* antibody titers were determined using an indirect immunofluorescent antibody test (IFAT) with *T*. *gondii* (RH) antigen, murine plasma diluted 2-fold from 1:25, and a fluorescein-isothiocyanate-labeled secondary antibody. Titers were calculated as the reciprocal dilution producing the last visible fluorescence on the IFAT. A cut-off titer of 50 was used to determine seroconversion to *T*. *gondii*.

Prepulse inhibition of the acoustic startle response was preformed as previously described [38]. Mice were placed in a startle chamber on a transducer, calibrated to 1 Newton to measure the startle response (Kinder Scientific, Poway CA). Each procedure consisted of a 5 min acclimation period followed by 65 stimulation trials presented in a pseudorandom order using a 70 dB background white noise. The first five and the last five stimulations were the 120 dB startle. The other 56 trials were presented in pseudo-random order and consisted of 10 trials of a 120 dB startle, and 10 trials each of a 72 dB prepulse, 74 dB prepulse and 78 dB prepulse (2 dB, 4 dB and 8 dB above background, respectively) 100 ms prior to the 120 dB startle, as well as 4 trials each of a 72 dB, 74 dB, and 78 dB prepulse alone and 4 trials of no stimulation. An average of 15 seconds separated each trial (between 13–17 seconds). The startle response was calculated as the average startle magnitude from the 10 120 dB trials presented in random order. PPI was calculated as the percent change between the average startle response alone and the average startle response after the prepulse. Acclimation was measured as the percent change between the first five startle tests and the last five startle tests. Following the PPI test, the weight of each mouse was recorded.

The emergence test, open field and novel object tests were performed similar to those described by [49]. This test was performed 3–4 days after PPI. Three separate open field chambers 25 cm x 25 cm with a gray floor and clear plexiglass walls and each equipped with a video camera attached to a computer were used to record animal behaviors. Each experimental mouse was first tested in the emergence test. The mouse was placed inside a stainless steel cylinder (10 cm deep and 6.5 cm in diameter) and video recorded for 20 min. A trained observer blind to genotype and treatment watched the video and scored the following behaviors: the latency to exit the cylinder, the number of exits from the cylinder to the open field, and time spent in the cylinder during each entry. An exit or entrance was defined as placement of all four paws into the open field or into the cylinder, respectively. A maximum of 300s was used for the latency to exit. Additionally, the total time spend inside the cylinder was calculated.

At the end of the emergence test, the mouse was placed in the center of the next open field chamber and recorded for 20 min. Distance traveled was measured using LimeLight, a video tracking software that tracks the movement of the center of the mouse. For analysis, the open field arena was divided into 16 squares (4X4 arrangement) and the time spent within the inner 4 squares (time in center) was measured. Following the open field test, the mouse was moved to the third open field chamber with a small plastic toy secured to the center of the open field. Distance traveled and time in the center of the open field was determined. Between each subject, the chambers were thoroughly cleaned and wiped down with 70% ethanol.

The bobcat urine test was conducted 3–4 days following the emergence/open field/novel object test. Using two of the open field chambers described above, each mouse was first placed in the center of the first open field chamber and recorded for 20 min. The mouse was then moved to the next open field chamber that contained a plastic receptacle secured to the floor of the chamber in each of the 4 corners with one that contained 50 μl of bobcat urine. Diagonal to the bobcat urine was 50 μl of rabbit urine and water was placed in the two receptacles in the remaining corners. Each mouse was recorded for 20 min. Following each test, the chambers were cleaned as described above. The location of the bobcat urine was changed for every test to avoid any possible bias within the open field chamber. Time spent in each of the 4 quadrants was measured and a percent calculated for the preceding open field test and the test containing bobcat urine. Comparisons were made between the percent time spent in the bobcat urine quadrant and the corresponding quadrant in the preceding open field test. This comparison accounts for any bias in the time spend among quadrants.

### Data Analysis

Data from behavioral tests prior to infection were analyzed using parametric analysis of variance (ANOVA) across genotype and sex with Fisher’s PLSD post hoc comparisons where appropriate. Post treatment data from emergence test, open field, novel object, and body weight were analyzed using ANOVA across treatment, genotype and sex. Fisher’s PLSD post hoc comparisons were used where appropriate. For the PPI data, the effects of treatment, genotype, sex and prepulse intensity were analyzed using ANOVA with repeated measures design. For bobcat urine avoidance, a paired t-test was used to compare, for each experimental group, the percent time spent in the bobcat urine quadrant and the corresponding quadrant in the preceding open field test. For comparison of effects of antibody titers and change in open field activity an ANOVA was calculated across the control, low, medium and high antibody categories followed by Fisher’s PLSD post hoc comparisons. All statistical analysis was performed using StatView Software. Results were considered statistically significance at p<0.05.

## Results

### Reduced Body Weight Due to the Nurr1 +/- Genotype

**Fig 1:**
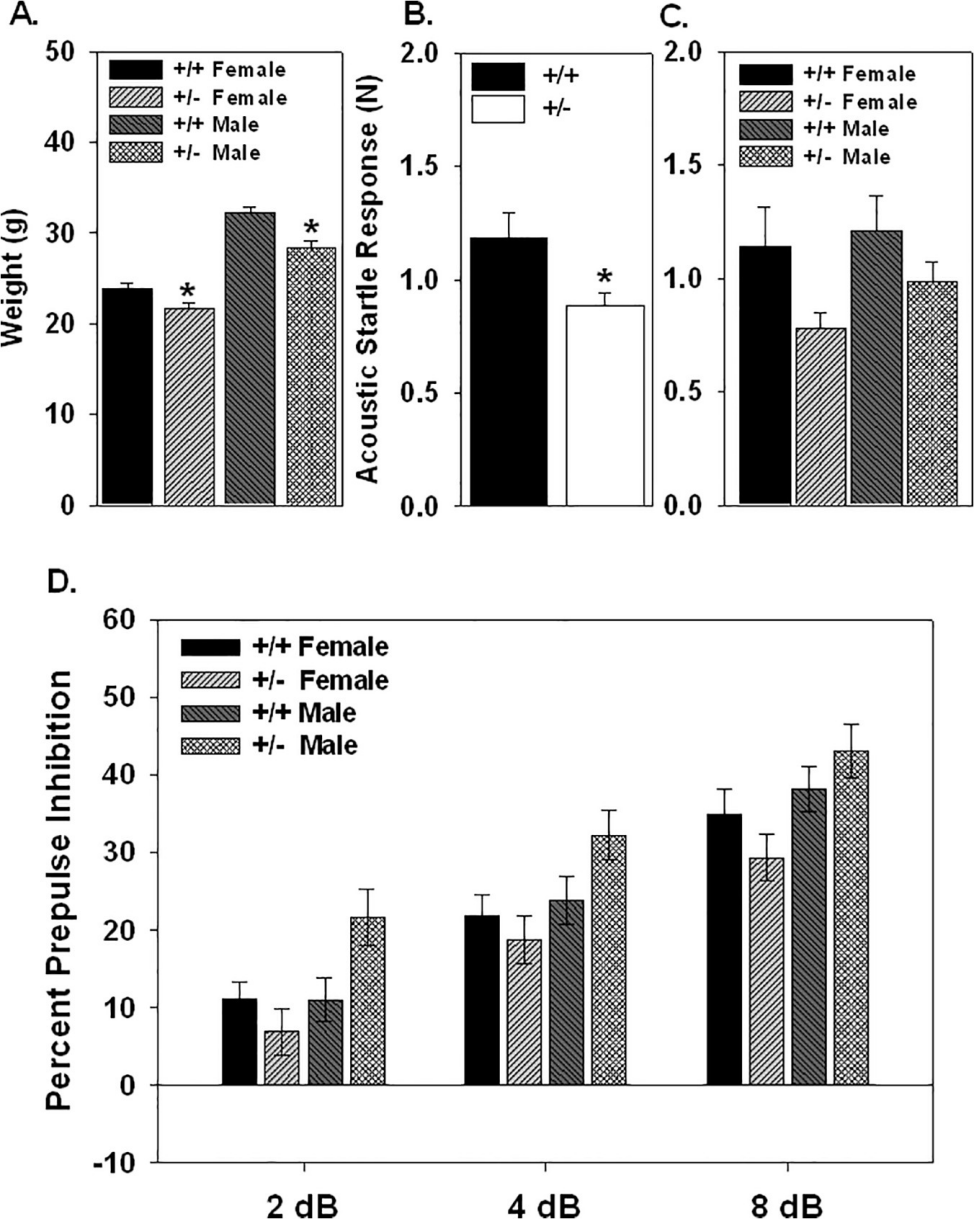
Body weight, acoustic startle response and prepulse inhibition of the acoustic startle response was measure in 120 day old female and male Nurr1 +/+ and +/- mice. Body weight (A.) was significantly reduced due to the +/- genotype and sex with significant reductions in both the female +/- (p = 0.037) and male +/- mice (p = 0.0002) as compared to +/+ mice. There was a significant reduction in acoustic startle response due to the +/- genotype (B.) that was not affected by sex (C.). No significant differences in percent prepulse inhibition of the acoustic startle response was found due to the +/- genotype or sex. Body weight and acoustic startle: *genotype difference p≤0.05 based on ANOVA with Fischer’s post hoc comparison. n = 19 female +/+; 21 female +/-; 29 male +/+; 18 male +/-.

A number of behavioral studies have been carried out on Nurr1 null mice [38–40,50,51]. Here we found that the +/- genotype significantly reduced body weight (F_1,85_ = 9.89, p = 0.0023). Female and male +/- mice had a lower body weight than their +/+ littermates based on the pretest weight at 120d of age with an 8.7% and 12.1% lower body weight, respectively (Fig 1A). This difference appears to be due to a reduction in weight gain with aging as weight gain was also reduced in the +/- control mice weighed 6 weeks later (see below).

### Prepulse inhibition in the Nurr1 +/- Mice

In the pretest, PPI and acoustic startle response data was analyzed across genotype, sex and prepulse intensity using a repeated measures ANOVA. A significant genotype effect was observed in the acoustic startle response where the +/- genotype resulted in a reduction in magnitude of the startle response (F_1,89_ = 5.33, p = 0.023; Fig 1B.). No significant interaction was found between genotype and sex (Fig 1C.). No difference in PPI was observed due to the +/- genotype in either males or females. There was an overall trend toward a sex effect (F_1,77_ = 3.51, p = 0.065) much of which was due to differences between male and female +/- mice (Fig 1D.). The results from the male mice are very similar to what was previously reported [38]. Although Vuillermot et al. reported significant reduction in PPI in +/- mice, testing was done during the dark phase of the light cycle as compared to the light phase done here [40]. No difference in habituation to the startle response based on genotype was observed (data not shown). Additionally, there were no differences in PPI, acoustic startle response or habituation in the pretest results between control mice later injected with a vehicle and treatment mice later injected with *T*. *gondii* (data not shown).

### Emergence Test, Open Field Activity and Novel Object in Nurr1 +/- Mice

In the emergence test, no significant differences in latency to exit the cylinder or time in the cylinder were found due to the +/- genotype (Table 1). The most obvious difference in the emergence test was that the +/- mice entered and exited the cylinder more frequently than the +/+ mice as shown by a greater number of exits (F_1,81_ = 9.335, p = 0.003). This effect was similar across male and female +/- mice.

**Table 1:** Emergence test latency, time in cylinder and total exits.

	Latency (s)	Time in Cylinder (s)	Exits
**Pretest**			
Female +/+ (n = 18)	40.40 ± 12.76	445.32 ± 104.93	13.63 ± 2.18
Female +/- (n = 19)	49.20 ± 17.55	372.12 ± 62.38	18.78 ± 1.68
Male +/+ (n = 29)	75.44 ± 16.89	448.32 ± 71.10	11.76 ± 1.29
Male +/- (n = 18)	57.97 ± 14.79	299.54 ± 40.68	18.00 ± 1.44
+/+	61.55 ± 11.56	447.13 ± 59.06	12.50 ± 8.01
+/-	53.46 ± 11.40	336.81 ± 37.63	18.39 ± 1.09*
**Control**			
Female +/+ (n = 9)	16.13 ± 4.23	226.98 ± 59.78	17.78 ± 1.15
Female +/- (n = 7)	13.19 ± 3.16	437.14 ± 137.08	19.25 ± 3.39
Male +/+ (n = 17)	10.11 ± 2.24	342.68 ± 57.95	15.59 ± 1.47
Male +/- (n = 10)	6.76 ± 2.03	223.81 ± 53.19	19.60 ± 3.08
+/+	12.20 ± 2.10	282.89 ± 39.52	16.21 ± 0.96
+/-	9.62 ± 1.91	316.83 ± 60.96	21.57 ± 2.27*
All Control	11.14 ± 1.46	297.15 ± 34.07	18.46 ± 1.15
***T*. *gondii* infection**			
Female +/+ (n = 5)	8.50 ± 2.86	129.74 ± 43.33	13.60 ± 3.61
Female +/- (n = 10)	15.85 ± 5.64	203.31 ± 42.36	21.10 ± 3.10
Male +/+ (n = 9)	14.89 ± 3.66	133.68 ± 40.49	13.56 ± 1.62
Male +/- (n = 8)	10.95 ± 2.48	137.07 ± 28.69	19.25 ± 4.32
+/+	12.60 ± 2.63	132.27 ± 29.24	13.86 ± 1.67
+/-	13.68 ± 2.29	173.87 ± 27.24	20.28 ± 2.51*
All *T*. *gondii* infection	13.21 ± 2.15	155.67 ± 19.99^#^	17.47 ± 1.67

*Genotype effect

# *T*. *gondii* infection effect after ANOVA and post-hoc comparison. p<0.05.

**Fig 2:**
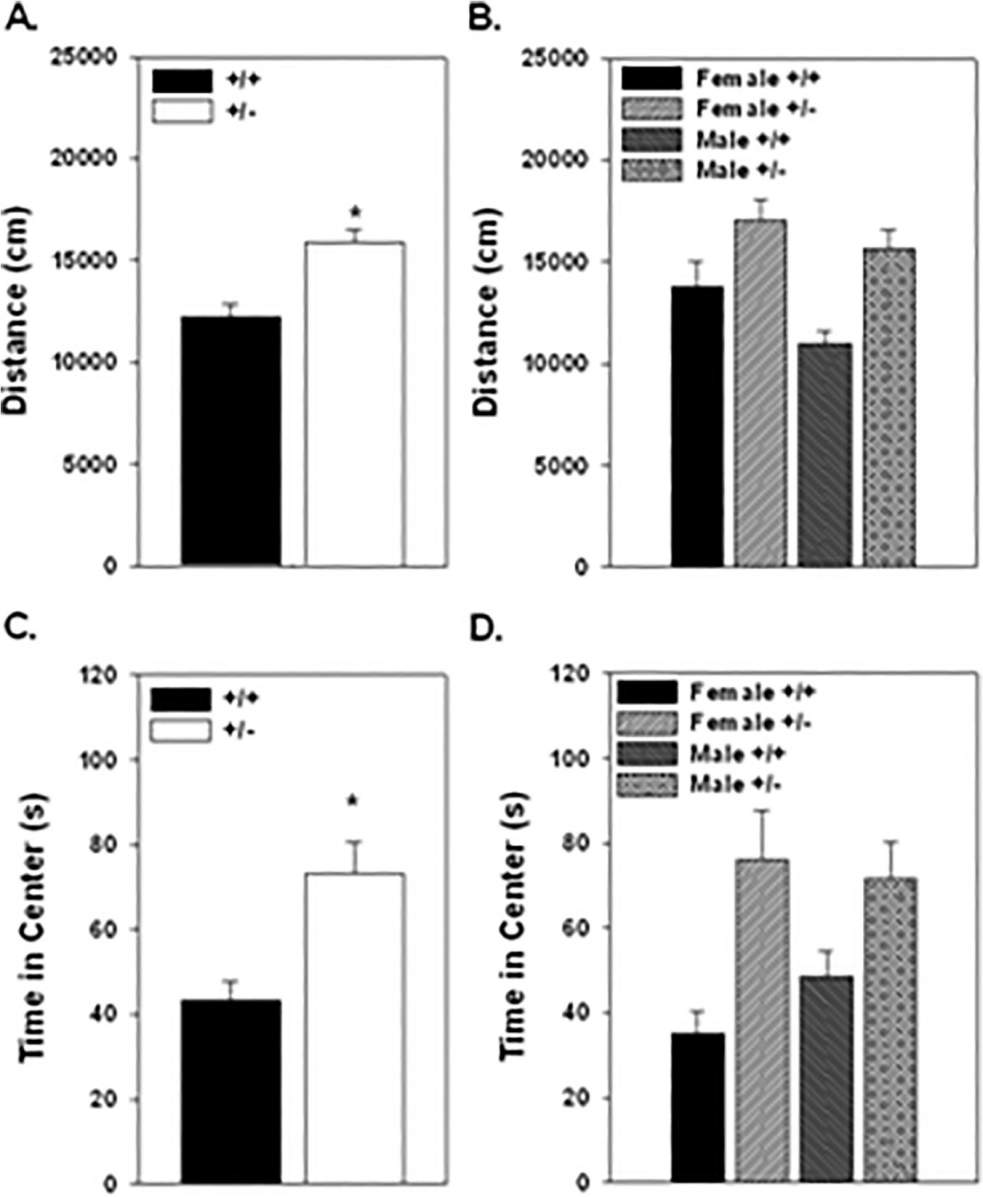
Behavior in an open field was tested in male and female Nurr1 +/+ and +/- mice. Total distance traveled (A., B.) and time in the center (C., D.) are shown based on genotype (A., C.) and genotype and sex (B., D.). The +/- mice were significantly more active than the +/+ mice as indicated by distance traveled, however, there was no significant interaction between genotype and sex (B.). Additionally, the +/- mice spent significantly more time in the center of the chamber than the +/+ mice (C.), which also showed no interaction between genotype and sex (D.). *significant difference based on ANOVA with Fischer’s post hoc comparison. n = 18 female +/+; 19 female +/-; 29 male +/+; 18 male +/-.

Parameters associated with open field activity analyzed included distance traveled and time in the center of the chamber. As previously reported, the +/- mice were significantly more active in the open field than the +/+ mice (F_1,80_ = 16.76, p = 0.0001; Fig 2A.). This was similar for both the male and female mice. Additionally, the +/- mice spent more time in the center of the open field chamber than the +/+ mice with similar effects in both the males and females (F_1,80_ = 15.17, p = 0.0002; Fig 2B.). No differences in the interaction with the novel object, as measured by time in the center of the chamber, were found due to the genotype (data not shown).

### Bobcat Urine Avoidance in the Nurr1 +/- Mice

**Fig 3:**
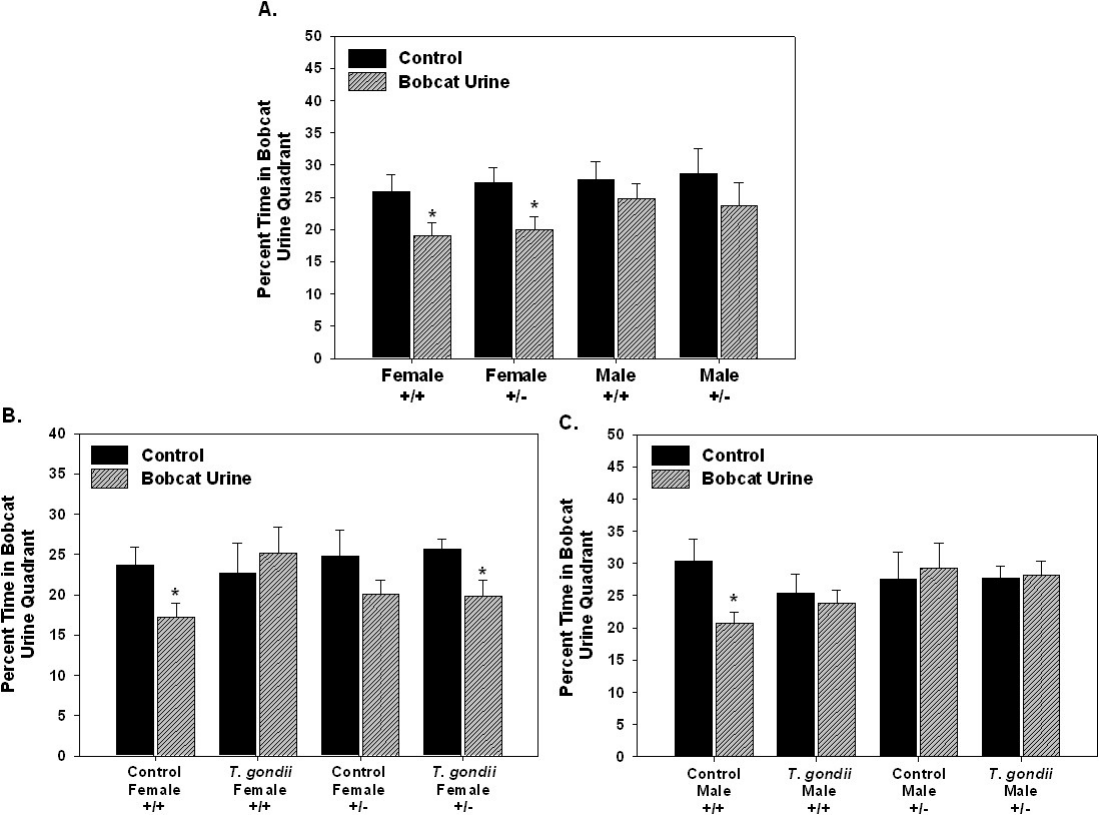
To demonstrate aversion to bobcat urine, percent time spent in the quadrant with bobcat urine (Bobcat Urine) was compare to the percent time spent in the same quadrant from the preceding open field test (Control) for each treatment group. In the first test (A.), female mice (both +/+ and +/-) showed significant aversion to bobcat urine as they spent significantly less time in the bobcat urine quadrant than in the corresponding quadrant from the open field test. No significant aversion was found using these parameters for the male mice. In the second test, after either a vehicle injection (Control) or *T*. *gondii* injection, the control female +/+ mice showed a significant aversion to bobcat urine that was abolished by injection with *T*. *gondii* (B). In contrast the control female +/- mice showed a trend toward aversion to bobcat urine but significant aversion to bobcat urine after *T*. *gondii* injection (B.). The control male +/+ mice showed significant aversion to bobcat urine that was also abolished by *T*. *gondii* injection (C.). In contrast, the male +/- mice showed no aversion to bobcat urine either in the control condition or after *T*. *gondii* injection. Comparisons based on paired t-test with p<0.05. Figure A: n = 18 female +/+; 21 female +/-; 29 male +/+; 18 male +/-. Figure B: n = 10 female +/+ controls; 6 female +/- controls; 5 female +/+ *T*. *gondii*; 10 female +/- *T*. *gondii*. Figure C: 17 male +/+ control; 10 male +/- controls; 10 male +/+ *T*. *gondii*; 8 male +/- *T*. *gondii*.

In the bobcat urine avoidance, time spent in each of the 4 quadrants of the open field was measure with bobcat and rabbit urine present and in the preceding open field test. The percent of time spent in the bobcat urine quadrant was compared to the percent of time spent in corresponding quadrant from the preceding open field test. There was a significant aversion to bobcat urine in the female mice (+/+, p = 0.036; +/-, p = 0.037; Fig 3A.). No significant effect of bobcat urine was found in the male mice. All pretest measures were also compared based on either later receiving the vehicle injection or *T*. *gondii* infection. No differences in any parameters were found in the groups prior to treatment (data not shown).

### Seroconversion to *T*. *gondii* antigen

A total of 86 mice were used to begin these experiments with 18 female +/+ mice, 21 female +/- mice, 29 male +/+ mice and 18 male +/- mice. Of those mice, data files were lost due to equipment malfunction in 2 emergence tests, 2 bobcat urine avoidance and 1 open field test. For *T*. *gondii* injection, 8 female +/+ mice, 13 female +/- mice, 12 male +/+ mice and 8 male +/- mice were injected. Three female +/+ mice, 3 female +/- mice and 3 male +/+ mice were seronegative for *T*. *gondii* and were excluded from the behavioral analysis. Additionally, 1 control female +/+ mouse died before the second round of behavioral tests.

**Fig 4:**
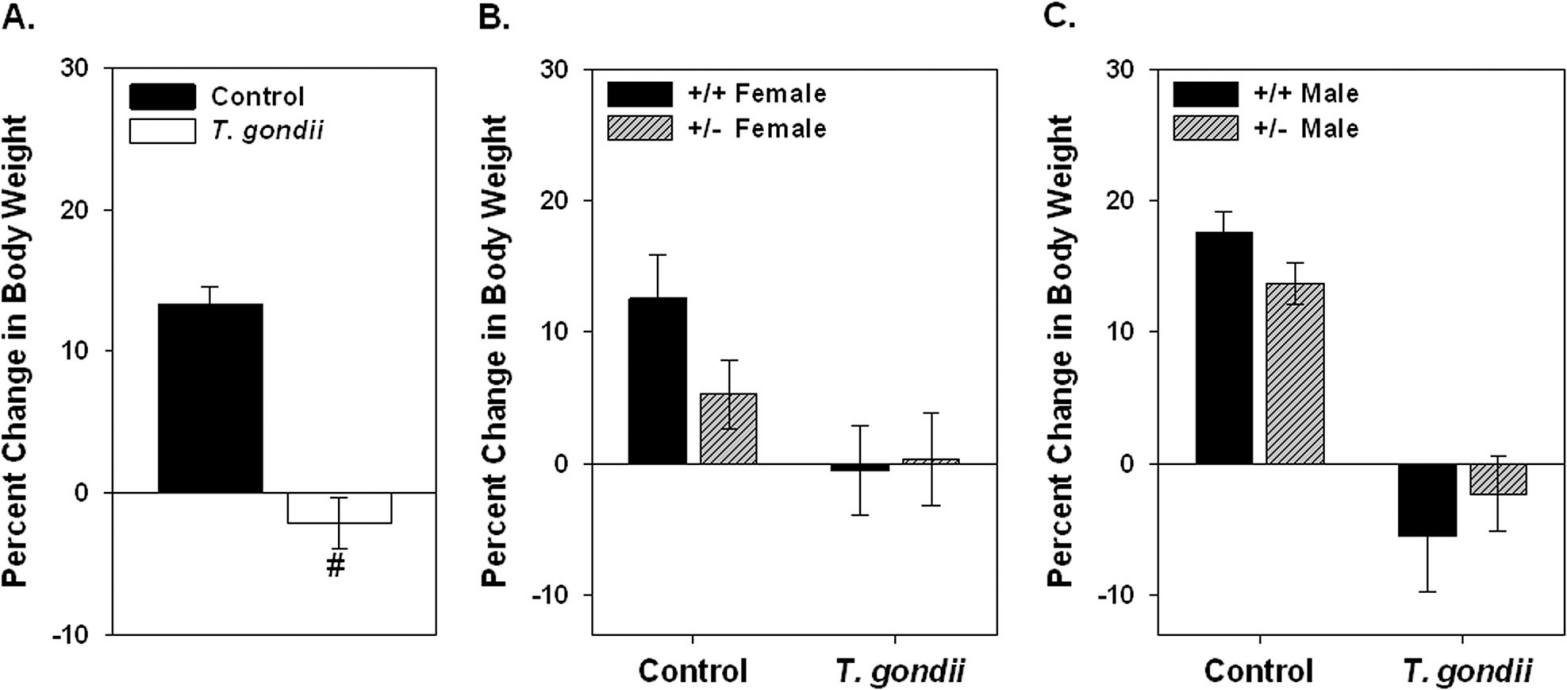
Effect of *T*. *gondii* infection on body weight is shown in female and male Nurr1 +/+ and +/- mice as percent change in body weight from weight prior to infection to body weight 6 weeks after infection or vehicle injection (Control). Infection with *T*. *gondii* significantly reduced weight gain (A.). There was no significant interaction in the change in body weight due to either the genotype or sex, however, the +/- mice gained less weight over this time (B., C.) and the infected male mice, on average, lost weight (C.) while the infected female mice showed no change in body weight (B.). #significant difference due to treatment based on ANOVA with Fischer’s post hoc comparison. n = 10 female +/+ controls; 9 female +/- controls; 5 female +/+ *T*. *gondii*; 10 female +/- *T*. *gondii*; 18 male +/+ control; 11 male +/- controls; 9 male +/+ *T*. *gondii*; 8 male +/- *T*. *gondii*.

Infection of mice was confirmed by seroconversion to *T*. *gondii* antigen. There was a wide range of antibody titers measured, with a titer of 50 as the lowest for inclusion as antibody positive, and up to 6400 measured in some mice. Geometric mean titers and range for seropositive mice based on sex and genotype are shown in Table 2. Nine mice injected with *T*. *gondii* had antibody titers below 50. These mice were excluded from the behavioral analysis. The +/- mice had an overall lower average antibody titer than the +/+ mice, but this was not statistically significant. Mice injected with *T*. *gondii* that seroconverted showed a significant reduction in weight gain (Fig 4A.). No significant interactions between *T*. *gondii* infection, genotype or sex on weight gain were found. Overall, there was a reduction in weight gain in female mice and net weight loss in the male mice (Fig 4B and 4C.). The female +/+ control mice gained 12.59±3.30% of their original body weight with the female +/- control mice gaining 5.26±2.58% of their body weight. In contrast, the +/+ female *T*. *gondii* infected mice averaged a reduction in the percent change in body weight with a -0.51±3.39% reduction in body weight. The +/- *T*. *gondii* injected female mice also had a lower percent change in body weight (0.32±3.50%). The *T*. *gondii* infected +/+ and +/- male mice had a -5.5±4.15% and -2.30±2.83% change in body weight as compared to 17.56±1.57% and 13.68±1.57% increase in body weight in male +/+ and +/- control mice, respectively.

**Table 2:** Antibody titers to *T*. *gondii*.

Sex-Genotype	Geometric mean titer	Range in titers for seropositive mice
Female +/+	919	50–3200
Female +/-	696.4	50–3200
Male +/+	1371.6	500–6400
Male +/-	1131.4	100–6400

### Effect of *T*. *gondii* infection on behavior – PPI, emergence test, open field and novel object

**Fig 5:**
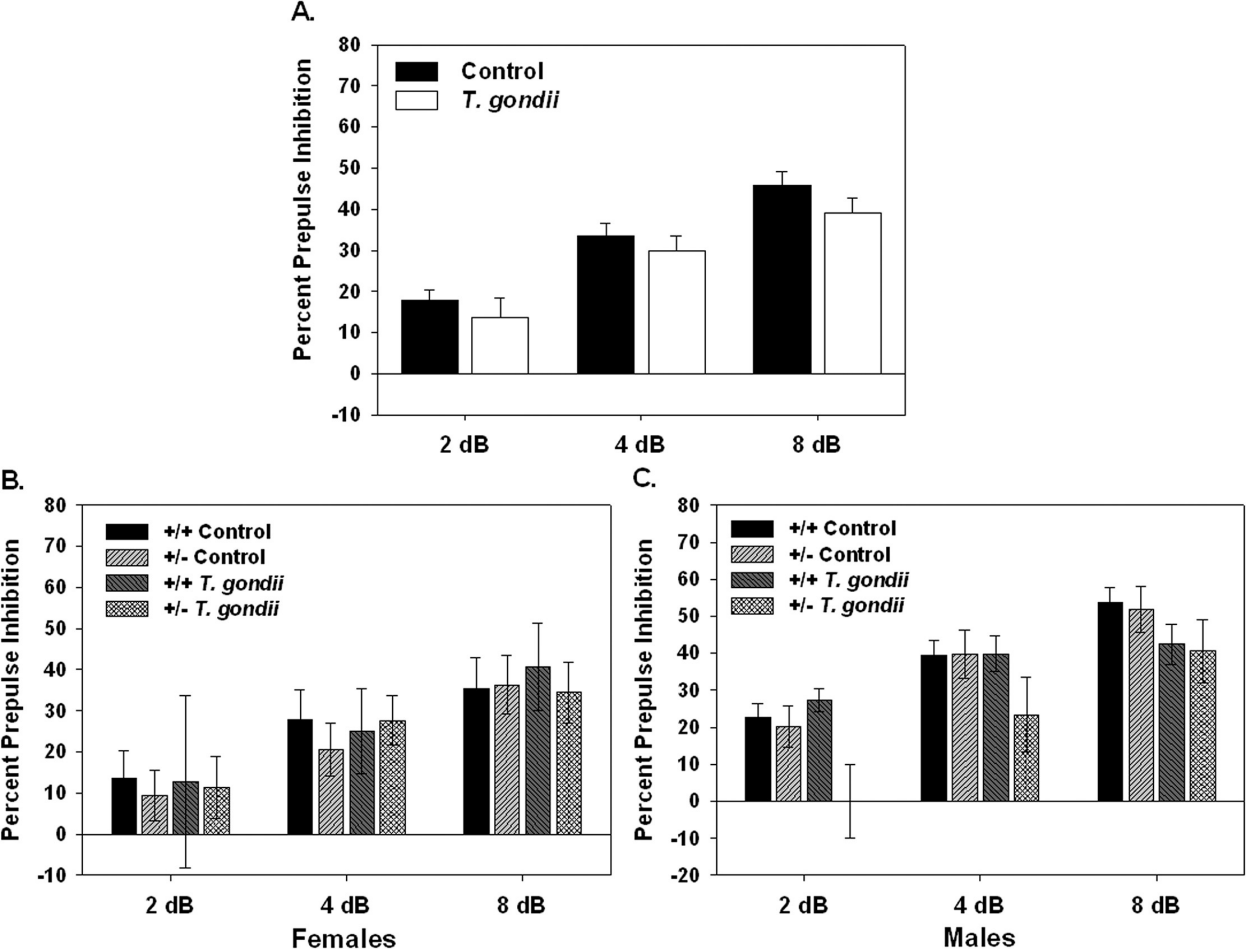
Percent prepulse inhibition in Nurr1 +/+ and +/- mice after vehicle injection (Control) or *T*. *gondii* infection (A.). Additionally, percent prepulse inhibition is show in female (B.) and male (C.) +/+ and +/- mice that received a vehicle injection (Control) or infection with *T*. *gondii*. *Toxoplasma gondii* infection had no significant effect on prepulse inhibition. There was a trend towards reduced percent prepulse inhibition in male +/- mice after chronic infection with *T*. *gondii* (C.). n = 10 female +/+ controls; 9 female +/- controls; 5 female +/+ *T*. *gondii*; 10 female +/- *T*. *gondii*; 17 male +/+ control; 12 male +/- controls; 9 male +/+ *T*. *gondii*; 7 male +/- *T*. *gondii*.

Infection with *T*. *gondii* had no effect on acoustic startle response or habituation of startle (data not shown). Additionally, no effect on PPI was found in the female mice after infection among any groups. In the male mice, *T*. *gondii* infection resulted in a decrease in PPI in the +/- mice as compared to both *T*. *gondii* infected +/+ mice and control +/- mice, particularly at the 2 dB prepulse intensity (Fig 5), however, this did not reach statistical significance.

No difference in latency to exit was found among groups in the emergence test performed six weeks after injection with *T*. *gondii* or vehicle (Table 1), however, latency to exit was significantly reduced in the post-injection test compared to the initial test. Infection with *T*. *gondii* did not affect latency to exit or total exits. *Toxoplasma gondii* infection significantly reduced the time spent in the cylinder (F_1,72_ = 9.18, p = 0.034) but had no effect on the latency to exit or number of exits.

**Fig 6:**
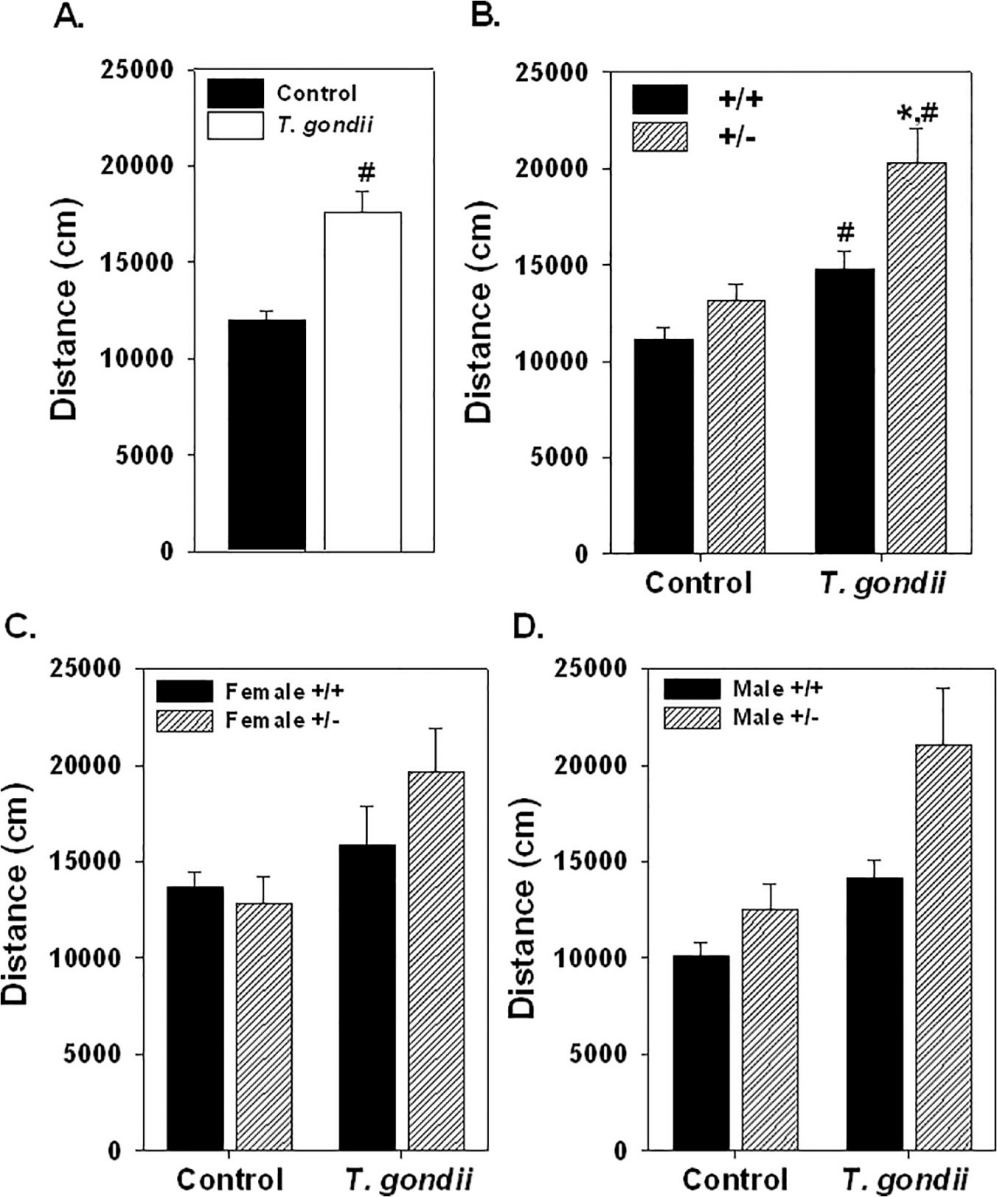
Open field activity is shown in female and male Nurr1 +/+ and +/- mice after *T*. *gondii* infection or vehicle injection (Control). Infection with *T*. *gondii* significantly elevated open field activity with all groups (sex and genotype) combined (A.). There was a significant interaction between infection with *T*. *gondii* and the Nurr1 +/- genotype on open field activity such that the +/- mice were significantly more active than infected +/+ mice and uninfected +/- control mice (B.). Both female and male +/- mice infected with *T*. *gondii* were more active in the open field than the control +/- mice (C., D.). Much of the increase in activity in the infected +/+ mice was the result of increased activity in the male mice. Significant genotype (*) and treatment (#) effects based on ANOVA with Fischer’s post hoc comparisons. n = 10 female +/+ controls; 8 female +/- controls; 5 female +/+ *T*. *gondii*; 10 female +/- *T*. *gondii*; 17 male +/+ control; 10 male +/- controls; 9 male +/+ *T*. *gondii*; 8 male +/- *T*. *gondii*.

**Fig 7:**
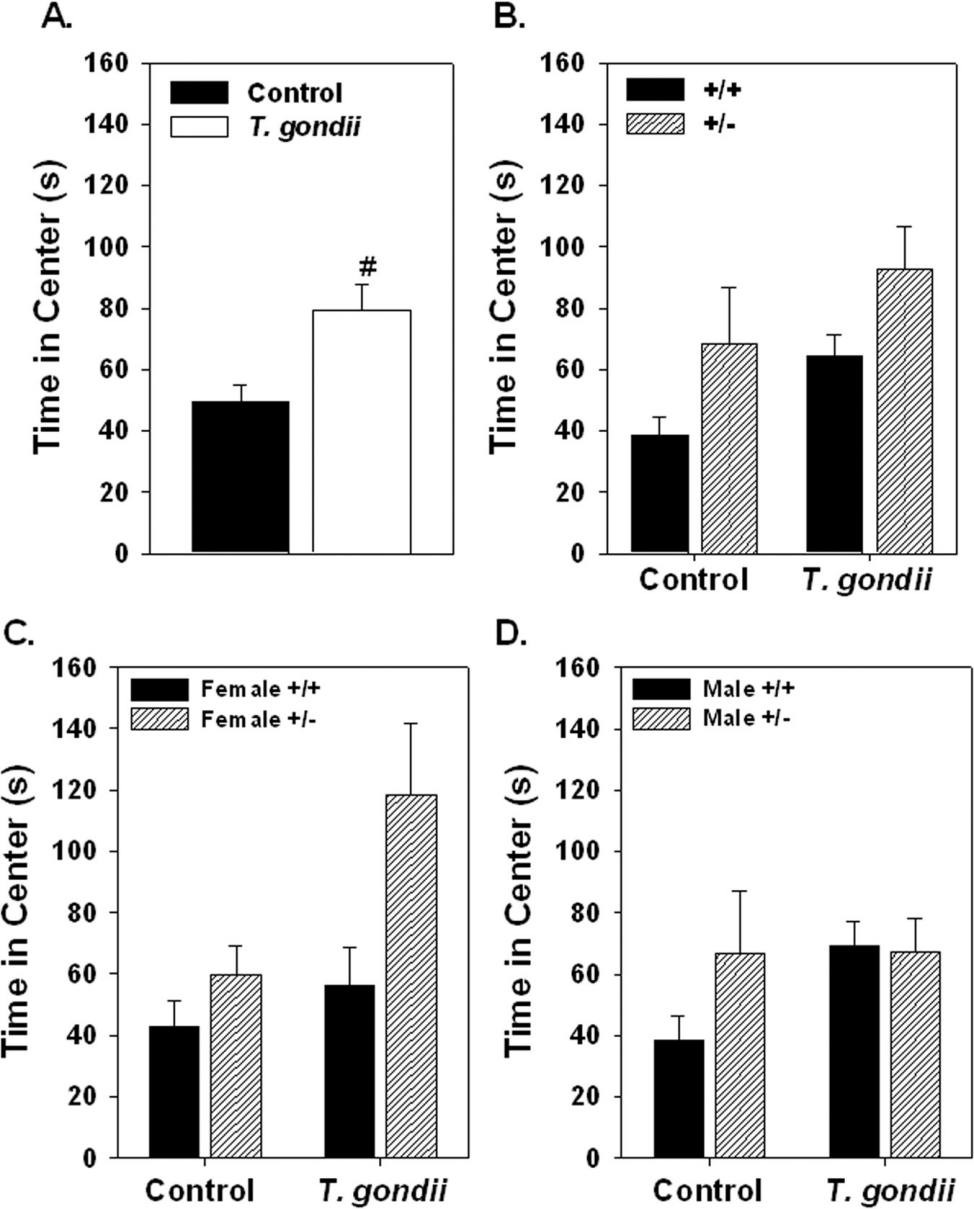
Time spent in the center of the open field is shown in female and male Nurr1 +/+ and +/- mice after *T*. *gondii* infection or vehicle injection (Control). Infection with *T*. *gondii* significantly elevated the time in the center of the open field with all groups (sex and genotype) combined (A.). Although, no significant interaction between *T*. *gondii* infection and genotype was found related to time in the center, much of the increase in time in center was the result of an increase time spent in the center for the +/+ mice. Significant treatment (#) effect based on ANOVA with Fischer’s post hoc comparisons. n = 10 female +/+ controls; 8 female +/- controls; 5 female +/+ *T*. *gondii*; 10 female +/- *T*. *gondii*; 17 male +/+ control; 10 male +/- controls; 9 male +/+ *T*. *gondii*; 8 male +/- *T*. *gondii*.

Infection with *T*. *gondii* significantly elevated the distance traveled in the open field (F_1,65_ = 6.56, p = 0.0031; Fig 6A.) and had a significantly greater effect on the +/- mice in elevating activity than on the +/+ mice (F_1,65_ = 4.573, p = 0.036; Fig 6B.). No effect of sex was found as female and male +/+ and +/- mice showed similar elevation of open field distance (Fig 6C and 6D.). Similarly, the time in the center was significantly elevated by infection with *T*. *gondii* (F_1,66_ = 8.407, p = 0.0056; Fig 7A.). No significant interactions were found with genotype or sex (Fig 7B, 7C and 7D.).

**Fig 8:**
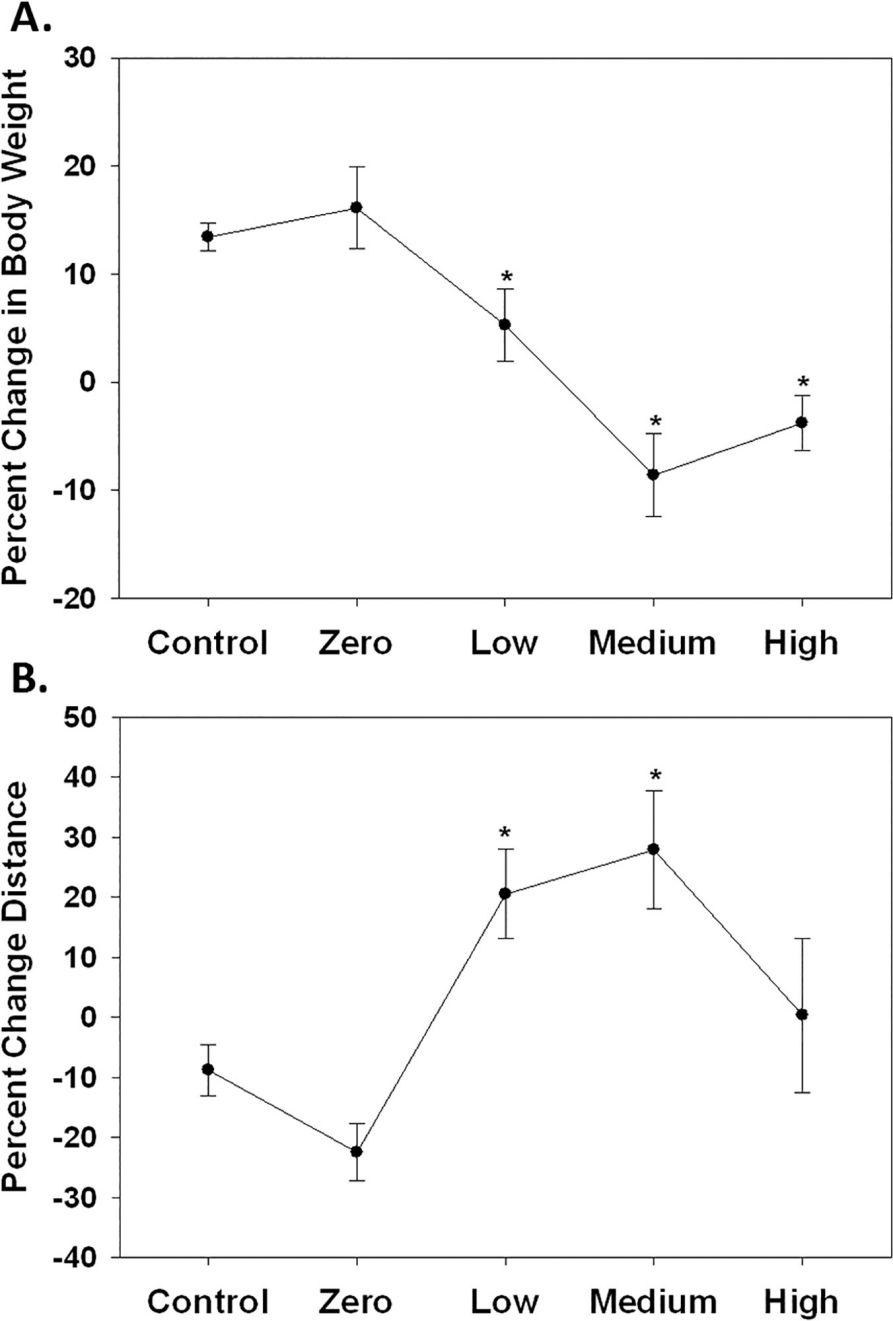
Percent change in body weight and change in distance traveled in the open field are shown in relation to the level of *T*. *gondii* antibodies. For this analysis, male and female Nurr1 +/+ and +/- mice are combined. Control refers to all mice injected with vehicle. Zero refers to mice injected with *T*. *gondii* but with no detectable antibody titers to *T*. *gondii*. The Low group consists of mice with antibody titers between 50 and 400. The Medium group consists of mice with antibody titers between 800–1600. The High group consists of the mice with antibody titers >3200. For body weight, there were significant reductions in body weight at all three antibody levels, Low, Medium and High (A.). For change in distance traveled in the open field, there was an inverted U-relationship consisting of a significant increase in distance traveled for mice in the Low and Medium antibody levels, but no significant effect on distance in mice at High antibody levels (B.). Significant antibody (*) effect based on ANOVA with Fischer’s post hoc comparisons. n = 47 Control; 4 Zero; 11 Low; 8 Medium; 11 High.

In order to understand the variables associated with changes in the behavior of mice infected with *T*. *gondii*, the relationship between reciprocal antibody titers with changes in body weight and open field activity were determined. Based on our measurements, antibody titers were categorized into low (50–400), medium (800–1600) and high (≥3200) levels and compared to percent change in body weight and percent change in distance traveled in the open field tests (Fig 8). All mice (male and female, +/+ and +/-) were combined for this analysis. Data from the mice injected with *T*. *gondii* that did not seroconvert (reciprocal antibody titers less than 50 and labeled “Zero”) were included in the graph but not in the statistical analysis. There was a significant effect of antibody levels on change in body weight (F_3,70_ = 19.842, p<0.0001) where body weight was reduced in all seropositive groups compared to control mice (Fig 8A.). A similar change in body weight was observed in mice injected with *T*. *gondii* that were seronegative (“Zero” group) and control mice. When the percent change in distance traveled in the open field between the pretest and the post-test was graphed based on antibody levels, an inverted U shaped distribution was found. Statistical analysis revealed a significant effect of antibody titer level and change in open field activity (F_3,70_ = 5.175, p = 0.0027). Low and medium antibody level groups had a significant increase in activity, however, activity of mice in the high reciprocal antibody level (≥3200) group was not different from control and appeared only slightly elevated (Fig 8B.). Mice that were injected with *T*. *gondii*, but were seronegative (Zero) showed a similar change in activity to the control mice.

### *T*. *gondii* infection, Nurr1 +/- genotype and Bobcat Urine Avoidance

In the bobcat urine avoidance test, there was a significant aversion to bobcat urine in both the male and female +/+ control mice, that was abolished in the *T*. *gondii* injected mice (Fig 3B and 3C.). In contrast, there was a trend toward aversion to bobcat urine in the control female +/- mice which became a significant aversion to bobcat urine in the *T*. *gondii* injected female +/- mice. The male +/- mice showed no aversion to bobcat urine as behavior was similar in control mice and *T*. *gondii* injected mice.

### Summary of Interactions between *T*. *gondii* Infection and the Nurr1 +/- Genotype

Collectively these behavioral data were consistent with those previously reported in the Nurr1 +/- mice with elevated exploratory behavior demonstrated by the greater exits during the emergence test and greater distance traveled in the open field. Additionally, the +/- mice showed reduced anxiety related behaviors consisting of decreased time spent in the cylinder in the emergence test and increased time spent in the center of the open field. *Toxoplasma gondii* infection produced effects in +/+ mice that mimicked some of the effects due to the +/- genotype such as a reduction in age related weight gain, elevated open field activity, and increased time spent in the center of the open field. Similarly, *T*. *gondii* infection alone had no effect on PPI just as the +/- genotype alone does not alter PPI. However, the combination of the +/- genotype and *T*. *gondii* infection had a cumulative effect, specifically seen as a greater effect on elevating open field activity. Additionally, preliminary evidence suggests that *T*. *gondii* infection and the +/- genotype may disrupt PPI in male +/- mice.

## Discussion

Understanding the genetic mutations that predispose individuals to schizophrenia and how these genetic components interact with environmental factors to lead to schizophrenia are important in order to provide interventions that could prevent the development of schizophrenia. Because infection with *T*. *gondii* is an important risk factor for developing schizophrenia, here we investigated how this infection may interact with the heterozygous deletion of the Nurr1 gene, which is necessary for dopamine neuron development, important for dopamine neurotransmission and also implicated to have a role in the development of schizophrenia [30–35].

The current investigation includes additional data on how the Nurr1 +/- genotype alters behavior and the interaction with a chronic, adult-acquired infection with *T*. *gondii*. A consistent behavioral consequence of the +/- genotype is an increase in seeking behavior as demonstrated by elevated activity in an open field. Previous studies have reported similar elevated open field activity in +/- male and female mice [39,40]. This effect is greatest with a novel open field or with a mild-stressor such as a vehicle injection [39,40,50]. Our data illustrates that repeated open field tests can eliminate the difference between +/- and +/+ mice as seen by the lack of difference in distance traveled between the vehicle injected +/- and +/+ mice. Also of note is that the +/- mice showed decreased anxiety related behavior based on a significant increase in the time in the center of the open field. These data from the open field and emergence test demonstrate that the Nurr1 +/- genotype has effects that are mostly independent of sex. Here we also found no significant effect on PPI between male +/+ and +/- or female +/+ and +/- mice, however, there was a trend towards differences in PPI between male and female +/- mice.

Attenuated Nurr1 expression, as occurs in the Nurr1 +/- mice, has been proposed as a rodent model for behaviors related to symptoms of schizophrenia based on elevated open field activity in these mice [38–40]. Open field activity is a behavior that is associated with dopamine neurotransmission because amphetamine and other dopamine stimulating drugs increase open field activity while dopamine D2 receptor antagonists have a strong effect on decreasing open field activity [50,52–54]. Additionally, transgenic mice with altered dopamine neurotransmission genes that increase dopamine neurotransmission, such as in the dopamine transporter or vesicular monoamine transporter 2 knockout/knockdown mice, show a profound elevation in open field activity [55–58]. The effects of the +/- genotype on open field activity are most likely due to dopamine neurotransmission as the need for Nurr1 in the development and continued survival of mesencephalic dopamine neurons has been demonstrated by gene deletion studies [30,32,33,59]. Additionally, the Nurr1 +/- genotype results in reduced expression of several dopamine neurotransmission genes and altered dopamine neurotransmission, which includes reduced tissue dopamine levels in the nucleus accumbens and elevated basal dopamine levels in the shell of the nucleus accumbens [48,50,60].

The current data revealed that *T*. *gondii* infection significantly elevated open field activity in both +/+ and +/- mice. This effect was exacerbated in the +/- mice. Time spent in the center of the open field was also significantly increased by *T*. *gondii* infection, but did not differ across genotypes. An increase in open field activity has been previously reported in adult mice chronically infected with *T*. *gondii* [23,24,26,61,62]. Other studies, however, have found either no effect on activity or even a decrease in activity after chronic *T*. *gondii* infection in mice [63–65]. Xiao et al. reported sex-specific effects on open field activity, with an increase in female mice and a decrease in male mice chronically infected with *T*. *gondii* [26]. Some of these differences may be due to differences in testing parameters. Specifically Skallova et al., who found a decrease in activity in infected female mice, used a dark arena with a red light, which reduces the anxiety related response to a bright open field [64]. Differences in infection routes and protozoal stages (either peroral introduction of cysts or parenteral injection of tachyzoites) and the strain of mice used could also contribute to these discrepancies. In general, data from the current study found that infection with *T*. *gondii* elevated open field activity and reduced anxiety related behaviors, as evidenced by distance traveled in open field test and time in the center of the open field, respectively. These data indicate that the +/- mice are especially susceptible to the effects of a chronic, adult acquired infection with *T*. *gondii*, specifically illustrated by effects on elevated open field activity and reduced anxiety related behaviors.

Avoidance of bobcat urine that was abolished by *T*. *gondii* infection was only found in the +/+ mice. Vyas et al. found that avoidance is dose dependent, such that an optimal amount of bobcat urine is necessary to demonstrate aversion and attraction, but it appeared the +/+ female mice were the most sensitive to this effect [66]. Interestingly, the female +/- mice showed significant bobcat urine avoidance after *T*. *gondii* infection while the male +/- showed no aversion to bobcat urine. These data show differences in susceptibility to aversion to bobcat urine, reversal of aversion with *T*. *gondii*, and effects on open field activity across the groups tested. This suggest that separate mechanisms may exist that underlie these behaviors. Additionally, these data show that genetic predisposition may influence the extent of altered behavior due to *T*. *gondii* infection in animals.

PPI is the decrease in startle response when the startle stimulus is preceded by a low intensity stimulus, which is a commonly used measurement of sensorimotor gating. Deficits in sensorimotor gating are consistently observed in schizophrenia patients and these deficits correlate with positive schizophrenia symptoms [41,42,44–47]. PPI is regulated by dopamine neurotransmission as systemic amphetamine and infusion of dopamine in the nucleus accumbens disrupts PPI and disrupted PPI can be normalized by dopamine receptor antagonists. Since PPI can be measured in similar ways between rodents and humans, PPI has become a commonly used model for investigating parameters associated with schizophrenia symptoms [67–70]. To date, no studies have demonstrated deficits in sensorimotor gating after infection with *T*. *gondii*. We corroborate these finding as we found no effect on PPI in the +/+ mice, however, when *T*. *gondii* was combined with +/- genotype in male mice, we found a trend toward disruption of PPI. The male +/- mice were also the group with the highest open field activity after *T*. *gondii* infection. This reduction in PPI, although not significant, was similar in magnitude to the significant reduction in PPI found in male +/- mice when isolated at weaning although the post-weaning isolation study had a larger sample size than the current one [38]. Elevated dopamine turnover and elevated amphetamine stimulated dopamine release was also found in isolated +/- mice, as compared to group raised +/- mice and isolate +/+ mice [48]. That isolated +/- mice were observed to have altered dopamine neurotransmission concurrent with disrupted PPI suggests that *T*. *gondii* is also enhancing dopamine neurotransmission in the +/- mice, causing altered PPI and elevated open field activity.

Since elevated open field activity is linked to elevated dopamine neurotransmission and *T*. *gondii* has been shown to express tyrosine hydroxylase enzymes leading to *in vivo* synthesis of dopamine, an attractive hypothesis is that *T*. *gondii* cysts in the brain directly elevate dopamine levels to alter these dopamine related behaviors [21,22]. This hypothesis is intriguing since abnormal dopamine neurotransmission has been implicated in schizophrenia and infection with *T*. *gondii* increases the risk for developing schizophrenia [28,71–76] Unfortunately, no studies to date have measured markers of dopamine neurotransmission, dopamine electrophysiology or determined if the dopamine produced by cysts is sufficient to impact overall extracellular or stimulated dopamine levels.

The problem with the dopamine hypothesis of chronic *T*. *gondii* infection for elevating the risk of schizophrenia is that alterations in dopamine neurotransmission in schizophrenia are considerably more complicated than simply an increase in tissue and/or extracellular dopamine levels [73]. Alterations in dopamine neurotransmission found in schizophrenia suggest that there is hypofunction in mesocortical dopamine neurotransmission, but elevated subcortical dopamine neurotransmission. Clinical PET studies reported a significantly greater dopamine response to amphetamine in the striatum, but a decrease in markers of dopamine innervations in the prefrontal cortex in patients with schizophrenia [77]. Presynaptic effects in dopamine neurons in schizophrenia include elevated dopamine synthesis and dopamine release in the striatum [78]. Furthermore, the super-sensitivity to dopamine, as demonstrated by the increased response to dopamine stimulating drugs such as amphetamine in patients with schizophrenia, is also associated with a dramatic increase in the high affinity state of the dopamine D2 receptors with only modest effects on overall dopamine receptor expression [29]. Currently, it is unclear how *T*. *gondii* affects the various parameters of dopamine neurotransmission across these different dopaminergic pathways.

Neuroinflammation, including infiltration of macrophages, activation of astrocytes and cuffing around vessels, is a consistent feature reported in the brains of rodents infected with *T*. *gondii* [23,63,79,80]. Also associated with these pathological processes are the release of inflammatory cytokines and the enhancement of the kynurenine pathway. Within astrocytes, tryptophan is degraded via this metabolic pathway to kynurenine which is further degraded to the neuroactive metabolite kynurenic acid [81]. Kynurenic acid is an endogenous antagonist to the α7 nicotinic acid receptor and the N-methyl-D-aspartate receptor [82–85]. Systemic kynurenine, which results in conversion to kynurenic acid in the brain, increases amphetamine stimulated dopamine release in the striatum, increases dopamine neuron activity, and disrupts PPI in rats [86–88]. Furthermore, ventral tegmental dopamine neurons are activated (i.e. increase firing rate and percent burst firing) by systemic kynurenine [89]. Infection with *T*. *gondii* has been shown to stimulate the kynurenine pathway and elevate kynurenic acid levels in the brains of mice [90,91]. Collectively, these observations provide a reasonable mechanistic hypothesis that infection with *T*. *gondii* alters dopamine related behavior via elevating kynurenic acid synthesis. Of note are the observations that kynurenic acid and kynurenine levels are elevated in the brains and cerebral spinal fluid of schizophrenia patients [92–97]. Additionally, anti-inflammatory drugs, such as cyclooxygenase inhibitors, have been shown to have some efficacy at treating schizophrenia symptoms [98,99]. Based on these data, inflammation and enhancement of kynurenic acid associated with *T*. *gondii* infection could be an important factor in mediating the changes in behavior found in rodent models and in contributing to an increase in the risk of schizophrenia.

A likely mechanism by which inflammation (i.e. cytokines, kynurenic acid etc.) could alter dopamine neurotransmission is by altering the function of dopamine neuron afferents. The regulation and control of mesoaccumbens dopamine neuron electrophysiology by afferents is well categorized. These areas include excitatory innervations from the pedunculopontine tegmentum that stimulate burst firing of these dopamine neurons and inhibitory innervations from the ventral pallidum neurons that regulate the number of spontaneously active dopamine neurons [98,99]. The ventral subiculum and prefrontal cortex can regulate the nucleus accumbens which has inhibitory input to the ventral pallidum [74]. Additionally, pyramidal neurons in the prefrontal cortex innervate the ventral tegmental dopamine neurons in such a way as to provide excitatory (glutamate) innervation to GABAergic interneurons that then synapse on dopamine neurons that project to the nucleus accumbens but direct excitatory innervation to dopamine neuron that have reciprocal innervate back to the prefrontal cortex [100]. Therefore, a condition that inhibits neuron output from the prefrontal cortex would produce elevated subcortical dopamine neurotransmission and attenuated cortical dopamine neurotransmission, reproducing the condition of subcortical hyperdopaminergic neurotransmission and cortical hypodopaminergic neurotransmission described in schizophrenia. Similarly, impairment in ventral pallidum neurons innervating mesoaccumbens dopamine neurons could increase the number of spontaneously active dopamine neurons, resulting in elevated dopamine neurotransmission and an increase in the number of dopamine neurons that can be induced into burst firing, which occurs in response to behaviorally relevant stimuli. Both of these conditions would result in elevated open field activity and possibly disrupted PPI.

Another possible mechanism by which inflammation could impact dopamine neurotransmission is a direct effect on dopamine neuron function. The predominant effect of cytokines on dopamine neurotransmission, however, is a reduction in dopamine neurotransmission and depression-like behaviors [101]. Since the behaviors measured in the current investigation occurred after chronic *T*. *gondii* infection, reduced dopamine neurotransmission early during the infection could set up a delayed condition of elevated dopamine neurotransmission via dopamine receptor super-sensitivity, for example [102].

A growing number of animal studies are demonstrating how a genetic predisposition can potentiate the effects of an environmental stressor [101,103–105]. Previously we found that post-weaning isolation, which is used to model the effect of early life stressors that increase schizophrenia risk, interacts with the Nurr1 +/- genotype. Here we find that another risk factor for schizophrenia, *T*. *gondii* infection, is also potentiated in Nurr1 +/- mice. The elevated susceptibility of the Nurr1 +/- mice to the altered behaviors resulting from *T*. *gondii* likely results from either, 1) dysregulation of the dopamine system and/or 2) increased propensity for inflammation due to a reduced or attenuated induction of Nurr1 levels. Nurr1 expression in dopamine neurons appears to be regulated by dopamine neuron activity and, as a transcription factor, Nurr1 has been found to regulate expression of various dopamine neurotransmission genes including dopamine transporter, vesicular monoamine transporter 2, GTP cyclohydrolase and tyrosine hydroxylase [60,106–108]. Collectively, these data, combined with the effects in the +/- mice, suggest that Nurr1 serves as an important regulatory step between dopamine neuron activity and subsequent expression and function of dopamine neurotransmission proteins. If *T*. *gondii* infection alters dopamine neuron activity, the regulatory response to this change would be different in the +/- mice. Post-weaning isolation alters the response in +/- mice resulting in an elevated amphetamine-stimulated dopamine release [48].

*Toxoplasma gondii* infection could exert effects on dopamine neurotransmission directly by the regulation of Nurr1 expression in neurons. A recent finding by Xiao et al. reported that acute infection with *T*. *gondii* resulted in an increase in expression of MiR-132, a cyclic AMP-responsive element binding regulated microRNA [109]. MiR-132 was also recently found to negatively regulate Nurr1 expression and inhibit the differentiation of embryonic stem cells into dopamine neurons [110]. If *T*. *gondii* increases MiR-132 in dopamine neurons resulting in repression of Nurr1 expression, then the +/- mice would be more vulnerable to attenuated Nurr1 expression, which would likely have a greater effect on dopamine neuron gene expression and dopamine neurotransmission. Since Nurr1 is also expressed in neurons in other brain regions, the effect of the +/- genotype combined with a possible direct effect of *T*. *gondii* on Nurr1 expression via MiR-132 could result in abnormal function of other brains regions, such as the ventral subiculum or prefrontal cortex.

Nurr1 expression and function is also regulated by and has a role in the downstream effects of inflammation. Specifically, Nurr1 expression was shown to be anti-inflammatory as reduced levels of Nurr1 in astrocytes enhance inflammation and neuronal degeneration resulting from lipopolysaccharide treatment [111]. This could potentially cause increased inflammatory responses in the Nurr1 +/- mice and make them more vulnerable to the effects of inflammation on dopamine neurotransmission. Collectively, these data provide several potential mechanisms through which chronic *T*. *gondii* infection can interact with the Nurr1 +/- genotype to precipitate susceptibility for disrupted dopamine related behaviors.

Another important finding in the current data is that a differential effect on open field activity was present with an inverted U relationship between activity and the level of humoral immune response based on antibody titers to *T*. *gondii*. This could be of considerable relevance to other studies on the behavioral effects of *T*. *gondii*. Previous studies using rodents did not report *T*. *gondii* antibody titers, only seropositivity to *T*. *gondii*. The current study found reduced weight gain after *T*. *gondii* infection which is similar to what was previously reported [63,65,112]. It is unclear the relationship between effects on weight and activity as a reduction in weigh could lead to greater activity or greater activity could produce weight loss. Although we found a relationship between weight and open field activity, there was not a significant correlation between change in weight and change in open field activity. Additionally, a change in body weight at all antibody titers was observed, whereas high antibody titers were not associated with an increase in activity.

These data suggest that the level of active (or humoral) immune response and antibodies produced to *T*. *gondii* could be an important indicator of the effects on behavior. This is of particular relevance as the increased risk of schizophrenia or severity of schizophrenia symptoms are related to levels of antibodies to *T*. *gondii*. Specifically, Brown et al. found an association between high IgG antibody titers (endpoint dilutions≥1:128) in the mother and increased risk of schizophrenia spectrum disorders (odds ratio of 2.6 when adjusted for maternal age) in their offspring [16]. Blomström et al., investigating risk of schizophrenia associated with IgG antibody levels in mothers to *T*. *gondii* in relation to the general population, found that antibody levels greater than the 75^th^ percentile was associated with significant increased risk of schizophrenia based on an odds ratio of 2.1 [15]. These authors also found an antibody-associated effect such that increased risk was related to antibody titers, with an odds ratio of 1.6 when antibody titers were between the 75^th^ and 90^th^ percentile and an odds ratio of 3.2 when antibody titers were greater than the 90^th^ percentile. Hinze-Selch found significantly higher T. *gondii* antibodies in patients with schizophrenia, although no differences in frequency of patients with schizophrenia and controls [113]. These levels of antibodies in human patients are likely equivalent to the low and medium levels in the murine groups from the current study. Although most previous studies tested for the presence of detectable antibodies to determine infection, none, to our knowledge, have compared behavioral effects and antibody titers, as has been done in human studies investigating risk of mental illness. Some of the differences in the effect of *T*. *gondii* infection on behavior, particularly open field activity, could be due to differences in the immune response [26,64,65]. If mice or rats had very high levels of antibodies, this could diminish the effects of *T*. *gondii* infection on behavior. Further investigation of antibody titers and behavior could provide additional insight into how *T*. *gondii* infection alters behaviors in rodents. In future studies, it will be interesting and prudent to investigate how additional parameters, such as other markers for immune activation including cytokine levels, could add to the predictive value of *T*. *gondii*-induced alterations on behavior. The use of both +/+ and +/- mice should enhance this modeling as we get a graded response with infection depending on genotype. Finally, direct effects of *T*. *gondii* antibodies remain a possibility as autoantibodies to several proteins in the brain have been described that result in changes in behavior and can lead to psychotic symptoms [114].

## Conclusions

These data demonstrate that reduced Nurr1 function, as found in the Nurr1 +/- genotype, makes mice more susceptible to *T*. *gondii* induced alterations in the dopamine related behaviors of open field activity. Additionally, the levels of immune response to *T*. *gondii* infection, particularly humoral response as measured by antibody titers to this protozoan, is not a linear relationship, but an inverted U relationship. This model reproduces the genetic-environmental hypothesis of schizophrenia where a specific gene mutation or environmental stressors alone are not sufficient to cause disease, however, the combination of the two are capable of causing the disease. Understanding the mechanism(s) through which chronic *T*. *gondii* infection alters behaviors in rodents and interacts with the Nurr1 +/- genotype could provide insight into how it can modify behaviors in humans and interact with other predisposing gene mutations to contribute to mental illnesses. Results from this study generate novel hypotheses and raise additional questions regarding the role of the immune system as related to *T*. *gondii* infection in the development of schizophrenia.
